# Clinicopathological features and risk factors analysis of lymph node metastasis and long-term prognosis in patients with synchronous multiple gastric cancer

**DOI:** 10.1186/s12957-021-02130-8

**Published:** 2021-01-21

**Authors:** Liang Chen, Chao Yue, Gang Li, Xuezhi Ming, Rongmin Gu, Xu Wen, Bin Zhou, Rui Peng, Wei Wei, Huanqiu Chen

**Affiliations:** Department of General Surgery, Jiangsu Cancer Hospital & Jiangsu Institute of Cancer Research & The Affiliated Cancer Hospital of Nanjing Medical University, Nanjing, 210009 Jiangsu Province China

**Keywords:** Synchronous multiple gastric cancer, Lymph node metastasis, Tumor recurrence, Prognosis, Risk factors

## Abstract

**Background:**

As a common malignancy, gastric cancer (GC) remains an important threat to human’s health. The incidence of synchronous multiple gastric cancer (SMGC) has increased obviously with technical advances of endoscopic and pathological examinations. Several studies have investigated the relationship between SMGC and solitary gastric cancer (SGC). However, little is known about the relationship between early and advanced SMGCs, and the independent risk factors of lymph node metastasis and prognosis in SMGC patients remain unclear.

**Methods:**

We retrospectively collected 57 patients diagnosed as SMGC and underwent radical gastrectomies from December 2011 to September 2019. Epidemiological data and clinicopathological characteristics of all patients were recorded. Postoperative follow-up was performed by telephone or outpatient service. Chi-squared test or Fisher’s exact test was adopted in analysis of categorical data. Continuous data were analyzed by using unpaired *t* test. Univariate and multivariate analyses were performed to investigate the independent risk factors of lymph node metastasis and tumor recurrence of SMGC.

**Results:**

There were 45 males and 12 females. The average age was 62.1 years old. There were 20 patients with early SMGC and 37 patients with advanced SMGC. Most of patients (91.2%) had two malignant lesions. Tumor recurrence occurred in 8 patients, among which 7 patients died from recurrence. The rates of total gastrectomy, tumor size ≥ 2 cm, poorly differentiated type, lymph node metastasis, ulcer and nerve invasion, and preoperative CEA level were significantly higher in advanced SMGC patients compared to those with early SMGC. Lymphovascular cancer plug and preoperative CA125 were the independent risk factors of lymph node metastasis in patients with SMGC. Lymph node metastasis, nerve invasion, and preoperative AFP might be the risk factors of tumor recurrence of SMGC, but need further validation.

**Conclusions:**

In patients with SMGC, the presence of tumor size ≥ 2 cm, poorly differentiated type, lymph node metastasis, ulcer, nerve invasion, and relatively high preoperative CEA level might indicate the advanced SMGC. More attention should be paid to lymph node metastasis in SMGC patients with lymphovascular cancer plug and high preoperative CA125. Lymph node metastasis, nerve invasion, and preoperative AFP might be associated with recurrence of SMGC, needing further validation.

## Background

Gastric cancer (GC) is remain an important threat to the human’s health, as it becomes the fifth most commonly diagnosed cancer and the third leading cause of cancer-related death worldwide [1]. Although the technical improvement of treatment, the prognosis of GC patients is still poor, especially for advanced GC.

With the technical advances of endoscopic and pathological examinations, the incidences of both early gastric cancer (EGC) and synchronous multiple gastric cancer (SMGC) have increased in the past decades [2, 3]. The proportion of SMGC has been reported to account for 4.8–20.9% of all GC patients in recent study [4]. Due to the relatively high incidence of SMGC, preoperative and intraoperative examinations should be performed meticulously to avoid missing the presence of SMGC. Epidemiologically, previous study showed that SMGC occurred more likely in the elderly, men, patients with family history of cancer, as well as the smokers and drinkers [3, 5, 6]. While histologically, SMGC often arise from gastric mucosa with chronic gastritis, atrophic gastritis, and especially severe intestinal metaplasia [5, 7]. The relationship between SMGC and solitary gastric cancer (SGC) was investigated in several previous studies. It showed that there was no significant difference of clinicopathological features and prognosis between EGC and early SMGC [8]. But a recent study demonstrated that male sex and submucosal invasion were the predictive risk factors of early SMGC [9]. Furthermore, the prognosis of patients with advanced SMGC was reported to be poorer compared to the SGC patients [6].

However, little is known about the relationship between early SMGC and advanced SMGC, and the independent predictive risk factors of lymph node metastasis (LNM) and prognosis in SMGC patients remain unclear. In order to provide theoretical basis for the evaluation of treatment and prognosis of SMGC, with this in mind, we conduct this study to investigate the correlationship of clinicopathological features between early SMGC and advanced SMGC, and evaluate the predictive risk factors of LNM and long-term prognosis in patients with SMGC.

## Materials and methods

### Patients

The details of cases were retrospectively collected from patients with confirmed SMGC and complete clinical data, who underwent radical gastrectomies in the General Surgery Department of the Affiliated Cancer Hospital of Nanjing Medical University from December 2011 to September 2019. In our study, SMGC was defined to be two or more malignant lesions simultaneously in the stomach confirmed by the postoperative pathological examinations. Furthermore, SMGC was diagnosed in accordance with Moertel’s criteria as follows: (1) each lesion must be pathologically confirmed to be malignancy, (2) all lesions must be clearly separated by the microscopically normal gastric wall, and (3) each lesion must be mutually isolated, rather than the consequence of local extension or metastatic tumor [10]. The exclusion criteria were as follows: patients with remnant gastric carcinoma, patients with synchronous malignant tumors of other organs, and patients with incomplete clinical data for analysis. A total of 57 patients with SMGC were ultimately enrolled in this study. All patients were informed of this study and signed informed consent. Our study was approved by the Ethics Committee of Affiliated Cancer Hospital of Nanjing Medical University.

### Data collection

Epidemiological data and clinicopathological characteristics of all patients were retrospectively recorded, including gender, age, surgical methods, body mass index (BMI), neoadjuvant chemotherapy, number of primary tumors, tumor size, histological type, tumor pT staging, ulcer, lymphovascular cancer plug, nerve invasion, preoperative tumor markers (containing CEA, CA19-9, AFP, CA153, CA125, and CA724), distant metastasis, operation time, postoperative hospital stay, postoperative complication, follow-up time, and long-term outcomes. Age was divided into < 65 years and ≥ 65 years groups according to age segmentation criteria recommended by the World Health Organization. Tumor size was defined according to the maximum diameter of the largest tumor lesion in the stomach. Histological type was regarded in terms of the poorer type of differentiation in the case of different histological types appeared between the lesions. Furthermore, tumor pT staging was defined according to the one with deeper invasion in the case of different depth of invasion displayed between the lesions. Levels of all tumor markers were tested preoperatively. BMI is calculated with the following formula: BMI = weight (kg)/height^2^ (m^2^).

### Postoperative follow-up

We performed the postoperative follow-up regularly by telephone or outpatient service. During this follow-up, all the patients were recommended for abdominal computed tomography (CT) scanning and gastroscopy examination. Abdominal CT scanning was performed every 6 months, and gastroscopy was performed every 12 months. Tumor recurrence, withdraw, and death of patients were recorded. Tumor recurrence was validated mainly by abdominal CT scanning and gastroscopic biopsy.

### Statistical analysis

Categorical variables were expressed as the frequency, and continuous variables were represented as the median (range). Categorical data were analyzed with chi-squared test or Fisher’s exact test by SPSS 24.0 software package. And the unpaired *t* tests by GraphPad Prism 8 software was used in the analysis of continuous data. Univariate analysis and log-rank test were performed to evaluate the influence factors of LNM and tumor recurrence, respectively. Multivariate analyses using binary logistic regression model and Cox regression model were adopted to validate the independent predictive risk factors of LNM and tumor recurrence. *P* value < 0.05 was regarded statistically significant.

## Results

### Clinicopathological characteristics of the patients

Of all 57 cases, 45 patients were male and 12 patients were female. The average age was 62.1 years old, ranging from 30 to 79 years. There were 32 cases in the < 65-year-old group, while 25 cases in ≥ 65-year-old group. In terms of surgical methods, 11 patients underwent Billroth I anastomosis, 9 patients received distal gastrectomy and Roux en-Y anastomosis, while 37 patients experienced total gastrectomy plus Roux en-Y anastomosis. Only 5 cases received neoadjuvant chemotherapy. Most of the patients (52 cases) were with two malignant lesions, while 3 cases and 2 cases were with three and four malignant lesions, respectively. Only 4 patients were with tumors < 2 cm, and 53 patients with tumors ≥ 2 cm. Histologically, 11 patients with well-differentiated type, and 46 patients with poorly differentiated type. Consistency of histology was positive in 33 cases. According to pTNM staging criteria, pT1 was regarded in 20 patients, 10 patients were defined as pT2, and 27 patients were regarded as pT3-T4. The consistency of tumor pT staging was positive in 27 cases. Ulcer presented in most of the patients (53 cases). Lymphovascular cancer plug and nerve invasion were detected positively in 13 and 14 cases, respectively. Distant metastasis appeared in 2 cases. Postoperative pathology showed that LNM occurred in 24 patients. There were 3 patients with postoperative complications, including anastomotic fistula, intraabdominal hemorrhage, and cardiac insufficiency.

Due to 15 patients (26.3%) were loss to follow-up, there were 42 patients who had the data of follow-up in this study. Tumor recurrence occurred in 8 patients (19.0%), among which 7 cases (16.7%) died from recurrence. The median of follow-up time was 27.5 months (ranging from 3 to 33 months) in patients with tumor recurrence, and it was 28.5 months (ranging from 1 to 91 months) in patients without recurrence.

### Comparison of clinicopathological features between early and advanced SMGC

**Fig. 1:**
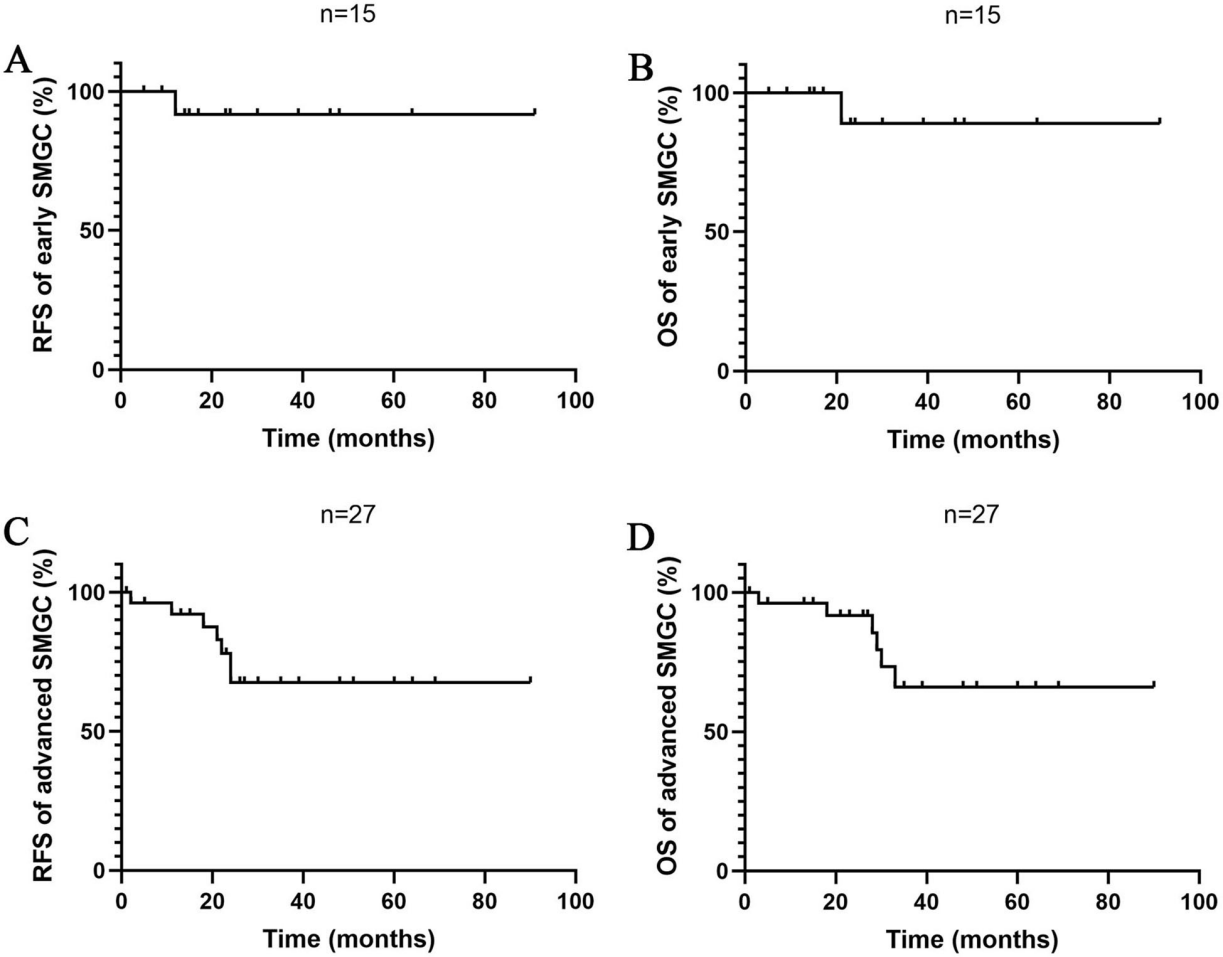
The RFS and OS curves of the early and advanced SMGC patients. **a** RFS of early SMGC. **b** OS of early SMGC. **c** RFS of advanced SMGC. **d** OS of advanced SMGC

**Fig. 2:**
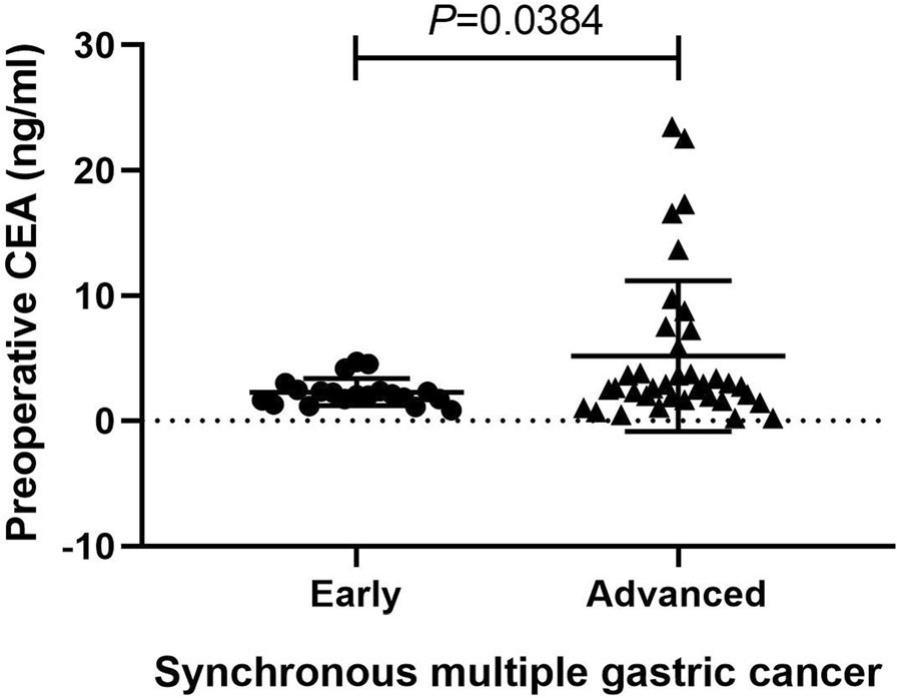
The preoperative CEA level in early SMGC patients was remarkably lower than that of patients with advanced SMGC (*P* = 0.0384)

In order to assess the difference of clinicopathological features between early and advanced SMGC, all patients were divided into early SMGC group (*n* = 20) and advanced SMGC group (*n* = 37). Fifteen patients had follow-up outcomes in early SMGC group, among whom one patient appeared tumor recurrence and then died. In advanced SMGC group, there were 27 patients with follow-up outcomes, among those 7 patients appeared tumor recurrence and 6 cases died from it. The RFS and overall survival (OS) curves of early and advanced SMGC patients were showed in Fig. 1. There were 11 and 9 patients underwent distal and total gastrectomies, respectively, in the early SMGC group, while most of the patients (28 of 37 patients) received total gastrectomy in the advanced SMGC group (*P* = 0.018). In the early SMGC group, 4 patients (20.0%) were < 2 cm of the tumor size, but no patient was < 2 cm in the advanced SMGC group (*P* = 0.012). Compared to 12 patients (60.0%) with poorly differentiated type in the early SMGC group, most of patients (91.9%) in the advanced SMGC group were the poorly differentiated type (*P* = 0.011). The occurrence rate of LNM was 15% (3/20) in early SMGC patients, significantly lower than that of 56.8% (21/37) in advanced SMGC patients (*P* = 0.002). Ulcer existed in most of patients with SMGC. Sixteen cases (80.0%) appeared ulcer in early SMGC patients, and it occurred in all the patients with advanced SMGC (*P* = 0.012). No nerve invasion appeared in the early SMGC group, while there were 14 patients (37.8%) with nerve invasion in the advanced SMGC group (*P* = 0.001). The median of preoperative CEA level in early SMGC patients was 2.08 ng/ml (range from 0.848 to 4.7 ng/ml), which was remarkably lower than 2.75 ng/ml (range from 0.2 to 23.5 ng/ml) in advanced SMGC patients (*P* = 0.0384) (Fig. 2) (Table 1).

**Table 1:** Comparison of clinicopathological features between early and advanced SMGC patients

Factors	Patients (*N*)	SMGC		*P* value
		Early	Advanced	
Gender				0.088^#^
Male	45	13	32	
Female	12	7	5	
Age (years)				0.121
< 65	32	14	18	
≥65	25	6	19	
Postoperative hospital stay, median (range, day)	–	12(9-18)	13(8-55)	0.298
Surgical methods				0.018*^#^
B I	11	8	3	
DG + R-Y	9	3	6	
TG + R-Y	37	9	28	
BMI, median (range, kg/m^2^)	–	21.5 (17.3–26.6)	22.3 (17.6–30.5)	0.2919
Hypertension				1.000^#^
Yes	12	4	8	
None	45	16	29	
Diabetes				0.607^#^
Yes	4	2	2	
None	53	18	35	
Number of primary tumors				0.100^#^
Two	52	18	34	
Three	3	0	3	
Four	2	2	0	
Tumor size				0.012*^#^
< 2 cm	4	4	0	
≥2 cm	53	16	37	
Histological type (Adenocarcinoma)^a^				0.011*^#^
Well-differentiated	11	8	3	
Poorly differentiated	46	12	34	
Consistency of histology				0.174
Positive	33	14	19	
Negative	24	6	18	
Lymph node metastasis				0.002*
Yes	24	3	21	
None	33	17	16	
Ulcer				0.012*^#^
Positive	53	16	37	
Negative	4	4	0	
Lymphovascular cancer plug				0.111^#^
Positive	13	2	11	
Negative	44	18	26	
Nerve invasion				0.001*^#^
Positive	14	0	14	
Negative	43	20	23	
Preoperative CEA, median (range, ng/ml)	–	2.08 (0.848–4.7)	2.75 (0.2–23.5)	0.0384*
Preoperative CA19-9, median (range, U/ml)	–	11.035 (0.6–43.2)	10.1 (1.28–173.6)	0.453
Preoperative AFP, median (range, ng/ml)	–	2.755 (0.947–4.61)	2.66 (1.05–8.25)	0.3063
Preoperative CA153, median (range, U/ml)	–	7.79 (3.44–20.6)	8.99 (4.9–20.82)	0.7443
Preoperative CA125, median (range, U/ml)	–	10.91 (5.51–19.51)	11.42 (3.28–39.09)	0.2492
Preoperative CA724, median (range, U/ml)	–	1.545 (0.2–8.35)	1.95 (0.699–141.1)	0.4754
Operation time, median (range, minute)	–	158 (90–262)	168 (102–340)	0.6577
Postoperative complication				0.545^#^
Yes	3	0	3	
None	54	20	34	
Recurrence				0.222^#^
Yes	8	1	7	
None	34	14	20	
Total	57	20	37	

Significant difference existed in several clinicopathological features between early and advanced SMGC patients, including surgical methods, tumor size, histological type, lymph node metastasis, ulcer, nerve invasion, and preoperative CEA

*SMGC* synchronous multiple gastric cancer, *BMI* body mass index, *B I* Billroth I anastomosis, *DG* distal gastrectomy, *TG* total gastrectomy, *R-Y* Roux en-Y anastomosis

^*^Statistically significant

^**#**^Fisher’s exact test

### Univariate analysis of influence factors of LNM

**Fig. 3:**
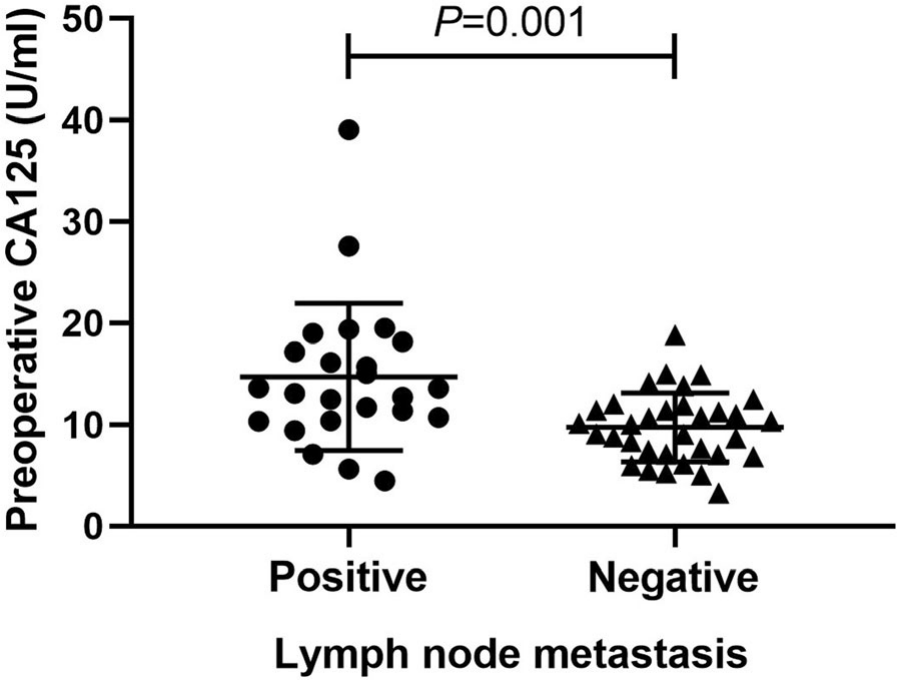
The preoperative CA125 level in patients with LNM was significantly higher than that of patients without LNM (*P* = 0.001)

According to the presence of LNM, all 57 patients with SMGC were divided into positive group (*n* = 24) and negative group (*n* = 33). Univariate analysis was performed to evaluate the influence factors of LNM of SMGC. Histologically, only one case (9.1%) of 11 patients with well-differentiated type was with positive LNM, and 23 cases (50.0%) of 46 patients with poorly differentiated type were with positive LNM. The incidence of LNM in patients with poorly differentiated type was significantly higher than that in patients with well-differentiated type (*P* = 0.017). In patients with pT1 and pT2, 3 cases (15.0%) of 20 patients and 3 cases (30.0%) of 10 patients were with positive LNM, respectively, while LNM was detected as positive in 18 cases (66.7%) of 27 patients with pT3-T4. Compared to patients with pT1 and pT2, the rate of LNM was obviously higher in patients with pT3–T4 (*P* = 0.001). The incidence of LNM in patients without lymphovascular cancer plug (27.3%, 12 of 44 patients) was significantly lower than that (92.3%, 12 of 13 patients) in patients with lymphovascular cancer plug (*P* = 0.000). Similarly, the rate of LNM was remarkably lower in patients without nerve invasion (27.9%, 12 of 43 patients) compared to the patients with nerve invasion (85.7%, 12 of 14 patients) (*P* = 0.000). In the positive group, the median of preoperative CA125 level was 13.355 U/ml (range from 4.46 to 39.09 U/ml), which was significantly higher than that of 10.05 U/ml (range from 3.28 to 18.87 U/ml) in the negative group (*P* = 0.001) (Fig. 3). It showed that histological type, tumor pT staging, lymphovascular cancer plug, nerve invasion, and preoperative CA125 level were the risk factors of LNM in patients with SMGC (Table 2).

**Table 2:** Univariate analysis of influence factors of lymph node metastasis in 57 patients with SMGC

Factors	Patients (*N*)	Lymph node metastasis		*P* value
		Positive	Negative	
Gender				0.2
Male	45	17	28	
Female	12	7	5	
Age (years)				0.426
< 65	32	12	20	
≥65	25	12	13	
Surgical methods				0.537^#^
B I	11	3	8	
DG + R-Y	9	4	5	
TG + R-Y	37	17	20	
BMI, median (range, kg/m^2^)	-	23.25 (17.8–30.5)	21.6 (17.3–29.7)	0.4048
Neoadjuvant chemotherapy				1.000^#^
Yes	5	2	3	
None	52	22	30	
Number of primary tumors				0.49^#^
Two	52	22	30	
Three	3	2	1	
Four	2	0	2	
Tumor size				0.13^#^
< 2 cm	4	0	4	
≥2 cm	53	24	29	
Histological type (Adenocarcinoma)^a^				0.017*^#^
Well-differentiated	11	1	10	
Poorly- differentiated	46	23	23	
Consistency of histology				0.548
Positive	33	15	18	
Negative	24	9	15	
Tumor pT staging^b^				0.001*^#^
pT1	20	3	17	
pT2	10	3	7	
pT3-T4	27	18	9	
Consistency of tumor pT staging				0.203
Positive	27	9	18	
Negative	30	15	15	
Ulcer				0.631^#^
Positive	53	23	30	
Negative	4	1	3	
Lymphovascular cancer plug				0.000*
Positive	13	12	1	
Negative	44	12	32	
Nerve invasion				0.000*
Positive	14	12	2	
Negative	43	12	31	
Preoperative CEA, median (range, ng/ml)	–	2.51 (0.476–16.59)	2.32 (0.2–23.5)	0.6811
Preoperative CA19-9, median (range, U/ml)	–	12.055 (1.28–173.6)	8.82 (0.6–43.2)	0.1131
Preoperative AFP, median (range, ng/ml)	–	2.73 (1.26–8.25)	2.71 (0.947–6.94)	0.2103
Preoperative CA153, median (range, U/ml)	–	9.79 (3.44–20.82)	8 (4.67–20.6)	0.8183
Preoperative CA125, median (range, U/ml)	–	13.355 (4.46–39.09)	10.05 (3.28–18.87)	0.001*
Preoperative CA724, median (range, U/ml)	–	1.985 (0.699–8.35)	1.56 (0.2–141.1)	0.4285
Distant metastasis				0.173^#^
Positive	2	2	0	
Negative	55	22	33	
Total	57	24	33	

It indicated that histological type, tumor pT staging, lymphovascular cancer plug, nerve invasion, and preoperative CA125 level were the significant risk factors of lymph node metastasis in patients with SMGC

*SMGC* synchronous multiple gastric cancer, *BMI* body mass index, *B I* Billroth I anastomosis, *DG* distal gastrectomy, *TG* total gastrectomy, *R-Y* Roux en-Y anastomosis

^*^Statistically significant

^**#**^Fisher’s exact test

### Multivariate analysis of the independent risk factors of LNM

Based on the outcomes of univariate analysis, histological type, tumor pT staging, lymphovascular cancer plug, nerve invasion, and preoperative CA125 were defined as the independent variables, and dummy variable was set in tumor pT staging. LNM was regarded as the dependent variable. Binary logistic regression was performed to validate the independent predictive risk factors of LNM. Compared with the patients without lymphovascular cancer plug, the risk of LNM increased significantly in patients with lymphovascular cancer plug (*P* = 0.004; 95%CI, 6.445~24782.173). The increase of preoperative CA125 level was significantly positively associated with the risk of LNM of SMGC (*P* = 0.007; 95%CI, 1.131~2.192). Multivariate analysis indicated that lymphovascular cancer plug and preoperative CA125 were the independent predictive risk factors of LNM in patients with SMGC (Table 3).

**Table 3:** Multivariate analysis of the independent risk factors of lymph node metastasis in patients with SMGC

Variables	*B*	S.E	Walds	df	*P* value	Exp(B)	95%CI of Exp(B)
Histological type	0.128	1.505	0.007	1	0.932	1.137	0.059~21.718
Tumor pT staging^a^	–	–	3.421	2	0.181	–	–
Tumor pT staging (1)	0.647	1.698	0.145	1	0.703	1.909	0.068~53.221
Tumor pT staging (2)	2.711	1.532	3.133	1	0.077	15.044	0.747~302.777
Lymphovascular cancer plug^b^	5.991	2.106	8.093	1	0.004*	399.662	6.445~24782.173
Nerve invasion	1.268	1.302	0.948	1	0.330	3.554	0.277~45.629
Preoperative CA125	0.454	0.169	7.236	1	0.007*	1.575	1.131~2.192
Constant	− 8.708	2.987	8.497	1	0.004	0.000	–

Multivariate analysis revealed that lymphovascular cancer plug and preoperative CA125 level were the independent risk factors of lymph node metastasis in patients with SMGC

*SMGC* synchronous multiple gastric cancer

^a^1 indicate “pT1”, 2 indicate “pT2”, 3 indicate “pT3-T4”, and taking 1 as the reference

^b^0 indicate “Negative”, 1 indicate “Positive”, and taking 0 as the reference

*Statistically significant

### Univariate analysis of influence factors of tumor recurrence

**Fig. 4:**
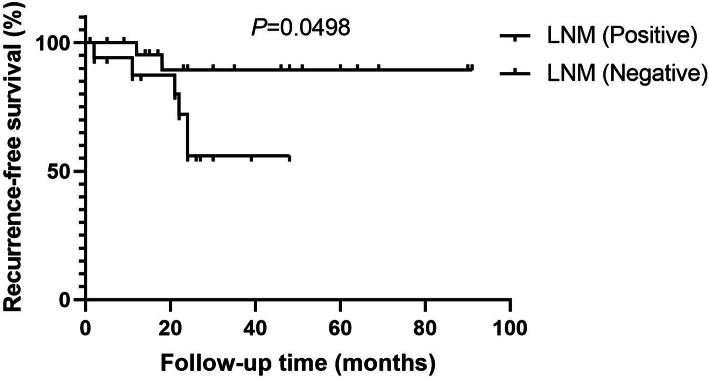
Log-rank test indicated that the difference of RFS was statistically significant between the patients with and without LNM (*P* = 0.0498)

Because of the cases of loss to follow-up, only 42 patients were ultimately included in survival analysis. According to the presence of tumor recurrence, all patients were separated into positive group (*n* = 8) and negative group (*n* = 34). Univariate analysis was used to investigate the influence factors of recurrence in SMGC patients. No significant difference of follow-up time existed between the positive and negative groups. There were 6 cases (35.3%) with tumor recurrence in 17 patients with LNM, and 2 cases (8.0%) with tumor recurrence in 25 patients without LNM. The incidence of recurrence was significantly higher in patients with LNM compared to those without LNM (*P* = 0.045). Four of 10 patients (40.0%) with nerve invasion had tumor recurrence, and 4 cases (12.5%) had tumor recurrence in 32 patients without nerve invasion. There was a trend that incidence of recurrence in patients with nerve invasion was obviously higher than that of patients without nerve invasion, but with no statistically difference (*P* = 0.075). The median of preoperative AFP level was 3.37 ng/ml (range from 1.18 to 8.25 ng/ml) in patients with tumor recurrence, tendentiously higher than 2.72 ng/ml (range from 0.947 to 5.92 ng/ml) in patients without recurrence, but no significant difference existed (*P* = 0.0791). Log-rank test showed that the difference of recurrence-free survival (RFS) was statistically significant between the patients with and without LNM (*P* = 0.0498) (Fig. 4). It revealed that LNM was the risk factor of tumor recurrence in patients with SMGC. However, nerve invasion and preoperative AFP might be the risk factors of recurrence, but without sufficient evidence (Table 4).

**Table 4:** Univariate analysis of influence factors of tumor recurrence in 42 patients with SMGC

Factors	Patients (*N*)	Recurrence		*P* value
		Positive	Negative	
Gender				0.369^#^
Male	32	5	27	
Female	10	3	7	
Age (years)				0.709^#^
< 65	23	5	18	
≥65	19	3	16	
Follow-up time, median (range, month)	–	27.5(3-33)	28.5(1-91)	0.1768
Postoperative hospital stay, median (range, day)	–	12.5(9-21)	12(8-55)	0.9009
Surgical methods				0.824^#^
B I	5	1	4	
DG + R-Y	7	2	5	
TG + R-Y	30	5	25	
BMI, median (range, Kg/m^2^)	–	21.6(19.5-27.8)	23.3(17.3-30.5)	0.6709
Neoadjuvant chemotherapy				0.479^#^
Yes	3	1	2	
None	39	7	32	
Hypertension				1.000^#^
Yes	8	1	7	
None	34	7	27	
Diabetes				1.000^#^
Yes	3	0	3	
None	39	8	31	
Number of primary tumors				0.158^#^
Two	38	6	32	
Three	3	1	2	
Four	1	1	0	
Tumor size				1.000^#^
< 2 cm	2	0	2	
≥2 cm	40	8	32	
Histological type (Adenocarcinoma)^a^				0.635^#^
Well-differentiated	8	2	6	
Poorly-differentiated	34	6	28	
Consistency of histology				1.000^#^
Positive	24	5	19	
Negative	18	3	15	
Invasive depth^b^				0.308^#^
T1	15	1	14	
T2	6	1	5	
T3-T4	21	6	15	
Consistency of invasive depth				0.697^#^
Positive	21	5	16	
Negative	21	3	18	
Lymph node metastasis				0.045*^#^
Yes	17	6	11	
None	25	2	23	
Ulcer				1.000^#^
Positive	38	7	31	
Negative	4	1	3	
Lymphovascular cancer plug				0.162^#^
Positive	8	3	5	
Negative	34	5	29	
Nerve invasion				0.075^#^
Positive	10	4	6	
Negative	32	4	28	
Preoperative CEA, median (range, ng/ml)	–	1.96 (0.476–22.59)	2.36 (0.2–23.5)	0.8142
Preoperative CA19-9, median (range, U/ml)	–	7.3 (1.28–32.85)	10.31 (1.63–32.82)	0.3112
Preoperative AFP, median (range, ng/ml)	–	3.37 (1.18–8.25)	2.72 (0.947–5.92)	0.0791
Preoperative CA153, median (range, U/ml)	–	10.22 (4.92–16.59)	8.51 (3.44–20.82)	0.9523
Preoperative CA125, median (range, U/ml)	–	10.2 (7.09–17.2)	11.145 (3.28–27.61)	0.8395
Preoperative CA724, median (range, U/ml)	–	1.81 (0.699–4.06)	1.61 (0.2–141.1)	0.6102
Operation time, median (range, minute)	–	167.5 (110–240)	166.5 (102–330)	0.8218
Distant metastasis				0.19^#^
Positive	1	1	0	
Negative	41	7	34	
Postoperative complication				0.479^#^
Yes	3	1	2	
None	39	7	32	
Total	42	8	34	

It revealed that lymph node metastasis was the risk factor of tumor recurrence in patients with SMGC. Nerve invasion and preoperative AFP level might be the risk factors of recurrence, but without sufficient evidence

*SMGC* synchronous multiple gastric cancer, *BMI* body mass index, *B I* Billroth I anastomosis, *DG* distal gastrectomy, *TG* total gastrectomy, *R-Y* Roux en-Y anastomosis

^*^Statistically significant

^**#**^Fisher’s exact test

### Cox regression analysis of the independent risk factors of tumor recurrence

According to results of univariate analysis, LNM, nerve invasion, and preoperative AFP were regarded as independent variables, and tumor recurrence was deemed as the dependent variable. Survival analysis of Cox regression was adopted to verify the independent predictive risk factors of recurrence in patients with SMGC. The increase of preoperative AFP level was tendentiously positively associated with the risk of tumor recurrence of SMGC patients, but with no significant difference (*P* = 0.081; 95%CI, 0.957~2.128). There was no significant difference of relationships between LNM or nerve invasion and risk of tumor recurrence. We found that preoperative AFP might be the independent risk factor of recurrence of SMGC patients, but need further validation (Table 5).

**Table 5:** Cox regression analysis of the independent risk factors for tumor recurrence in SMGC patients

Variables	*B*	S.E	Walds	df	*P* value	Exp(B)	95%CI of Exp(B)
Lymph node metastasis^a^	0.646	1.002	0.415	1	0.519	1.907	0.268~13.590
Nerve invasion^b^	0.794	0.806	0.969	1	0.325	2.211	0.455~10.734
Preoperative AFP	0.356	0.204	3.039	1	0.081	1.427	0.957~2.128

Cox regression analysis indicated that preoperative AFP might be the independent risk factor of recurrence in patients with SMGC

*SMGC* synchronous multiple gastric cancer

^a^0 indicate “None”, 1 indicate “Yes”, and taking 0 as the reference

^b^0 indicate “Negative”, 1 indicate “Positive”, and taking 0 as the reference

## Discussion

The treatment of GC, one of the most commonly malignancies, remains a long-term and difficult challenge worldwide. The prognosis of EGC has improved obviously with the technical advances of diagnosis and endoscopic dissection in past decade [3]. Due to the increased morbidity of SMGC which resulted from improvement of endoscopic technology recent years, more studies about SMGC patients are needed to enhance the understanding of SMGC.

As previous studies reported, elderly, male sex, tumor size ≤ 2 cm, and atrophic gastritis were the independent risk factors of occurrence of early SMGC, and a family history of GC, smoking, and alcohol consumption might be the risk factors of morbidity of patients with early SMGC [2, 3]. Therefore, we also chose 2 cm as the cutoff value for grouping of tumor size. Nitta et al. and Eom et al. demonstrated that age ≥ 65 years, male, a family history of cancer, tumor in the upper third of the stomach, early T stage, and severe intestinal metaplasia were the independent risk factors of developing SMGC [5, 11]. Compared to youngsters, atrophic change and intestinal metaplasia were more common in the gastric mucosa of elderly people, which might result in the higher morbidity of SMGC in elderly [3]. In the present study, similar to the outcomes of previous studies, most (78.9%, 45/57) of all patients with SMGC were male. However, only 43.9% (25/57) of the patients were with age ≥ 65 year-old, and just 7.0% (4/57) of the patients were with tumor size < 2 cm, which were differ from previous studies. Furthermore, the data of atrophic gastritis and intestinal metaplasia were partly lacking in our study. Insufficient sample size is the main limitation of this study.

As a special cohort of GC, SMGC is more common in EGC patients compared to advanced GC patients [2, 12]. Differently, in this study, only 35.1% of the patients were early SMGC, and most patients presented the advanced cancer lesions. In regard to the number of primary lesions, Zhao et al. reported that most patients presented two lesions, and three or more lesions existed in a few patients with SMGC [2]. Similar to previous study, 52 patients had two lesions, 3 patients presented three lesions, and only 2 patients were with four lesions in our study. And the number of primary lesions was not significantly associated with risk of LNM and tumor recurrence of SMGC. What is more, there was no significant difference of number of primary lesions between early and advanced SMGC patients.

As previous study confirmed, most main and minor lesions in SMGC patients were confined to the same third of the stomach, and the lower third of the stomach was the most common tumor location [2]. In patients with early SMGC, the clinicopathologic features were similar between main and minor lesions, including tumor location, macroscopic appearance, histological type, and invasion depth [12, 13]. However, we found that 42.1% (24/57) and 52.6% (30/57) of all patients with SMGC had inconsistent histological type and tumor pT staging, respectively, between the main and minor lesions. But the inconsistency of histological type or tumor pT staging was not significantly associated with LNM and recurrence of SMGC, and no significant difference of them existed between early and advanced SMGCs.

Previous study showed that the clinicopathologic characteristics and risk of LNM of early SMGC patients were not significantly different from that of early SGC patients [14]. Furthermore, there was no significant difference of long-term survival outcomes between patients with early SMGC and early SGC [2, 3, 15]. However, few previous studies have evaluated the correlationship between early and advanced SMGCs, and the independent risk factors of LNM and long-term prognosis in SMGC patients.

The rate of LNM, about 35.6% (67/188) in patients with SMGC, was reported to be significantly lower than that of patients with SGC [16]. Similarly, in our study, the incidence of LNM in SMGC patients was 42.1% (24/57). Tumor size ≥ 3 cm and lymphovascular invasion were confirmed to be the independent risk factors of LNM in patients with early SMGC [14]. Furthermore, lymphatic tumor invasion was regarded as the strongest predictor for LNM in EGC patients [17]. However, few studies were found to investigate the risk factors of LNM in SMGC patients. Similar to previous studies, lymphovascular cancer plug was proved to be the independent risk factors of LNM for SMGC patients in this study. No previous study reported the correlation between CA125 level and LNM of SMGC. Innovatively, we found that preoperative CA125 was significantly positively correlated with LNM in SMGC patients. Our results may will be significant in preoperative assess of LNM of SMGCs, but need further validation by a prospective study with larger sample size. Furthermore, histological type, tumor pT staging, and nerve invasion might be the influence factors of LNM, but with no significant difference.

With regard to the long-term prognosis, previous study indicated that the 5-year survival rate in patients with SMGC was significantly higher than that in patients with SGC [16]. Furthermore, LNM, serosal invasion, and curative resection were the independent prognostic factors of survival in SMGC patients [16]. Differently, there was a trend that LNM, nerve invasion, and preoperative AFP level might be the independent risk factors of a tumor recurrence of patients with SMGC in the present study, but with no statistically significant difference.

In conclusion, there were several factors with significant difference between early and advanced SMGC patients. In patients with SMGC, the presence of tumor size ≥ 2 cm, poorly differentiated type, LNM, ulcer, nerve invasion, and relatively high preoperative CEA level might make them more likely to be advanced SMGC, which should be paid more attention by surgeons. Furthermore, the appearance of lymphovascular cancer plug and high preoperative CA125 level indicated the increased risk of LNM in SMGC patients. Although with no significant difference, LNM, nerve invasion, and preoperative AFP level might be the predictive factors of recurrence of SMGC. A larger sample prospective study is needed to validate or improve the present outcomes because of the limitation of insufficient sample size in this study.

## Data Availability

The data and materials are available by contacting the authors.

## Abbreviations

GC: Gastric cancer
EGC: Early gastric cancer
SMGC: Synchronous multiple gastric cancer
SGC: Solitary gastric cancer
BMI: Body mass index
CT: Computed tomography
LNM: Lymph node metastasis
RFS: Recurrence-free survival
OS: Overall survival
B I: Billroth I anastomosis
DG: Distal gastrectomy
TG: Total gastrectomy
R-Y: Roux en-Y anastomosis
